# Coronavirus disease 2019 (COVID-19)-associated brain abscesses caused by *Pseudomonas aeruginosa* and *Aspergillus fumigatus*: two case and a review of the literature

**DOI:** 10.1186/s13256-023-04206-3

**Published:** 2023-12-04

**Authors:** Zeynab Yassin, Armita Farid, Sayedali Ahmadi, Maziar Emamikhah, Omid Motamedi, Mohammadamin Jafari, Azadeh Goodarzi

**Affiliations:** 1 Antimicrobial Resistance Research Center, Institute of Immunology and Infectious Diseases, School of Medicine, Iran University of Medical Sciences, Tehran, Iran; 2 School of Medicine, Iran University of Medical Sciences, Bisotun Street, 6.1 Alley, No 56, Tehran, 1431644311 Iran; 3 Department of Neurosurgery, Rasool Akram Medical Complex, School of Medicine, Iran University of Medical Sciences, Niayesh Street, Sattarkhan Ave, Tehran, 1431644311 Iran; 4 Department of Radiology, Rasool Akram Medical Complex, School of Medicine, Iran University of Medical Sciences, Niayesh Street, Sattarkhan Avenue, Tehran, 1445613131 Iran; 5 Department of Dermatology, Rasool Akram Medical Complex Clinical Research Development Center (RCRDC), School of Medicine, Iran University of Medical Sciences, Niayesh Street, Sattarkhan Avenue, Tehran, 1445613131 Iran; 6 Skin and Stem Cell Research Center, Tehran University of Medical Sciences, Tehran, Iran

**Keywords:** *Aspergillus fumigatus*, *Pseudomonas aeruginosa*, Brain abscess, COVID-19, Case series

## Abstract

**Background:**

Bacterial and fungal superinfections are commonly reported in patients with coronavirus disease 2019.

**Case presentation:**

We report the first case of brain and intramedullary abscesses caused by *Pseudomonas aeruginosa* and a rare case of brain abscesses caused by *Aspergillus fumigatus* in two post-coronavirus disease 2019 patients. The first patient—34-year-old Iranian woman—presented with weakness of the left upper limb, headaches, and lower limb paresthesia. She had a history of undiagnosed diabetes and had received corticosteroid therapy. The second patient—45-year-old Iranian man—presented with right-sided weakness and had a history of intensive care unit admission. Both patients passed away despite appropriate medical therapy.

**Conclusion:**

The immune dysregulation induced by coronavirus disease 2019 and its’ treatments can predispose patients, especially immunosuppressed ones, to bacterial and fungal infections with unusual and opportunistic pathogens in the central nervous system. *Pseudomonas aeruginosa* and *Aspergillus fumigatus* should be considered as potential causes of brain infection in any coronavirus disease 2019 patient presenting with neurological symptoms and evidence of brain abscess in imaging, regardless of sinonasal involvement. These patients should get started on appropriate antimicrobial therapy as soon as possible, as any delay in diagnosis or treatment can be associated with adverse outcomes.

## Introduction

The coronavirus disease 2019 (COVID-19) pandemic caused by severe acute respiratory syndrome coronavirus 2 (SARS-CoV-2) has affected over 505 million people and had caused 6.2 million deaths worldwide by 23 April 2022 [1]. Bacterial and fungal superinfections are increasingly reported in respiratory viral infections and have been related to increased morbidity and mortality [2–4].

Emerging evidence suggests that the number of bacterial superinfections in patients with COVID-19 is rising [5, 6]. Previous studies have proposed that the epithelial damage and immune dysregulation caused by COVID-19 can facilitate adhesion and invasion of the bacteria [3, 7]. *Klebsiella *spp., methicillin-resistant *Staphylococcus aureus* (MRSA), *Escherichia coli*, *Enterobacter *spp., *Streptococcus pneumoniae*, and *Pseudomonas aeruginosa* have all been isolated from patients with COVID-19 [8, 9].

*Pseudomonas aeruginosa* (*P. aeruginosa*) is a Gram-negative bacteria that commonly causes nosocomial infections in immunocompromised patients and patients with structural lung disease [10]. Biofilm production results in higher antimicrobial resistance and allows chronic colonization of *P. aeruginosa* in the host [11]. An increased abundance of *P. aeruginosa* has been found in the nose of patients with COVID-19, which was positively associated with viral RNA load [12]. *P. aeruginosa* has also been isolated from endotracheal tube secretions and blood cultures of patients with COVID-19 [9]. In this article, we report the first case of COVID-19-associated brain abscess caused by *P. aeruginosa.*

Fungal coinfections, including aspergillosis, mucormycosis, candidiasis, histoplasmosis, and cryptococcosis, have been widely reported among patients with COVID-19. These infections involve different organs, such as the lungs, heart, and brain [4, 13]. The suggested factors that predispose patients with COVID-19 to invasive fungal infections include immune dysregulation, lymphopenia, inflammatory state, corticosteroid use, intubation and mechanical ventilation, broad-spectrum antibiotic use, and indwelling catheters [14, 15]. *Aspergillus *spp. are known to cause invasive and life-threatening infections in immunocompromised patients [16]. The most frequently reported *Aspergillus *spp. infection in patients with COVID-19 is COVID-19-associated pulmonary aspergillosis (CAPA) [17, 18]. A review study showed that the incidence of CAPA was 15.1% among intensive care unit (ICU)-admitted patients with COVID-19 and that it was associated with increased mortality. Corticosteroids and immunosuppressant drugs conferred the highest risk for aspergillosis in these patients [19]. A few studies have also reported cases of extrapulmonary aspergillosis in patients with COVID-19 [20, 21]. In this article, we report a case of post-COVID-19 brain abscess caused by *Aspergillus fumigatus*.

## Case presentation 1

A 34-year-old Iranian woman presented to our hospital with progressive weakness of the left upper limb (started 10 days earlier with a wrist drop) and severe, sharp, nonpulsatile headaches accompanied by dizziness lasting 3–4 hours for 2 days. She also mentioned lower limb paresthesia 2 weeks before presentation. She had a history of COVID-19 infection a month prior to the presentation that was medically treated at home with remdesivir and corticosteroid injections, followed by oral prednisolone. She reported no fever, vomiting, or seizures and had no history of trauma or injection drug use. Neurological examination revealed an afebrile and oriented woman with decreased motor force in the left upper limb (3/5 proximally and 1/5 distally), a bilateral sensory level at T4, a left upward plantar reflex, and a mildly ataxic gait.

Initial laboratory results showed a normal leukocyte count (8500/µl, normal 4000–10,000), thrombocytopenia (97,000/µl, normal 140,000–440,000), elevated aminotransferases (ALT 171 IU/l, normal 5–40 and AST 113 IU/l, normal 5–40), increased lactate dehydrogenase (LDH 524 U/l, normal 225–500), elevated fasting blood sugar (FBS 201 mg/dl, normal 70–100), elevated hemoglobin A1c (7.4%, normal 3–6), normal erythrocyte sedimentation rate (ESR < 16 mm/h, normal < 20) and normal C-reactive protein (CRP < 6 mg/l, normal < 6).

**Fig. 1:**
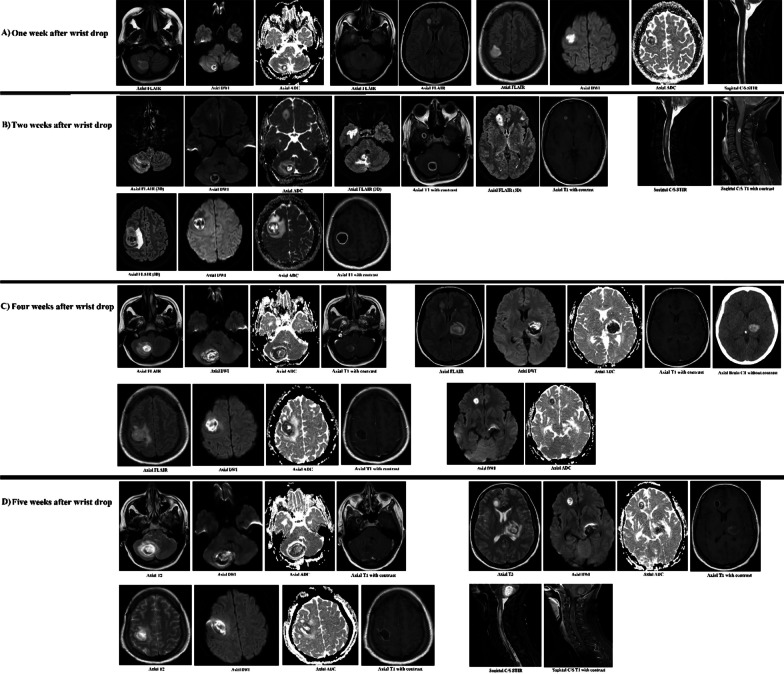
Brain magnetic resonance imaging (MRI) and brain computerized tomography (CT) of the patient with *Pseudomonas aeruginosa* brain abscesses (case 1). **A** Brain MRI without contrast performed 1 week after left upper limb weakness emergence (wrist drop) and before hospital admission shows multiple round brain lesions with high signal in T2-fluid attenuation inversion recovery (FLAIR) sequences and diffusion restriction in diffusion-weighted imaging (DWI) and apparent diffusion coefficient (ADC) sequences. The lesions in the right parietal and right cerebellum have the greatest sizes. In the last plane from the left, midsagittal short tau inversion recovery (STIR) MRI of the cervical spine (C/S), there is a longitudinal signal change in the cervical cord. **B** Brain MRI with contrast 2 weeks after wrist drop shows an enlargement of all the lesions shown in section A, all of them demonstrating a regular ring enhancement in contrast-enhanced-T1 MRI compatible with abscess. Note the considerable vasogenic edema surrounding the lesions as well as the round diffusion restriction of almost all the lesions in favor of pus collection. The longitudinal edema of the cervical cord is also enlarged in STIR with a small ring enhancement in the contrast-enhanced T1 MRI. **C** Four weeks after wrist drop and 2 weeks after admission the patient developed right hemiplegia. There is a round hyperdense hematoma in the left thalamus in brain CT corresponding with a high signal round lesion in FLAIR and low signals in DWI and ADC (blooming artifact in low B value) with peripheral vasogenic edema. If you look closely at section B, there is a very small hypersignal lesion with a faint ring enhancement in this location altogether in favor of hemorrhage in a small abscess in the left thalamus. All other abscesses are also increased in size compared with section B. **D** MRI performed 5 weeks after wrist drop, a few days before the patient’s death. All the abscesses are enlarged and there is a recollection of pus material in the right parietal and cervical cord abscess, which were drained surgically one week earlier

Computerized tomography (CT) scan of the head revealed multiple round hypodense lesions with rim enhancement in the right frontal lobe (10 × 12 mm), left frontal lobe (10 × 10 mm), and right parietal lobe (23 × 28 mm) with surrounding vasogenic edema in favor of multiple brain abscesses. Paranasal sinuses were normal. Magnetic resonance imaging (MRI) of the head also showed multiple ring-enhancing round lesions with peripheral edema and with central diffusion restriction in both cerebral hemispheres consistent with brain abscesses. Abscesses had internal small foci of blooming artifact from blood product and hemorrhage. Cervical MRI revealed an intraaxial mass with peripheral enhancement and peripheral edema in favor of abscess formation at the level of C2–3 (Fig. 1A, B). CT scan of the chest showed multilobar peripheral ground-glass opacities in favor of resolving COVID-19 infection. Abdominal ultrasound, which was performed because of elevated aminotransferases, showed increased parenchymal echo of the liver suggestive of grade 1 fatty liver disease, and a round 5 mm lesion in the right kidney suggestive of angiomyolipoma.

Empiric therapy was started with vancomycin, ceftriaxone, metronidazole, cotrimoxazole, and liposomal amphotericin B. Blood cultures, sputum smear and culture for tuberculosis, rheumatologic panel, viral hepatitis panel, HIV antibody, and COVID-19 polymerase chain reaction (PCR) test came back negative. Serology results (IgM and IgG) for *Toxoplasma gondii* were positive. Two weeks after admission, the patient developed sudden worsening of the headache, and dysarthria, and facial asymmetry and right-sided weakness emerged. Repeated brain CT scan and MRI showed an increase in the number and size of the previous lesions, with new abscesses in the left thalamus with secondary hemorrhage (Fig. 1C). Ceftriaxone and metronidazole were discontinued, and meropenem was added to the antibiotic regimen.

The patient was scheduled for a stereotactic biopsy and drainage of the parietal lobe brain abscess and the intramedullary cervical abscess. Drained fluid from the abscesses was sent for microbiological evaluation, which revealed a moderate number of polymorphonuclear (PMN) cells, but no organisms. Despite appropriate medical therapy, the patient’s neurological symptoms did not improve, and she became increasingly lethargic. One month after admission, the patient entered cardiac arrest and could not be resuscitated. The brain MRI that was performed a few days before her death had shown an increase in the size of previous lesions as well as recollection of pus in the drained abscesses of the right parietal lobe and cervical spine (Fig. 1D).

Eventually, PCR of the brain abscess fluid revealed *Pseudomonas aeruginosa* as the culprit organism for the patient’s condition.

## Case presentation 2

A 45-year-old Iranian man presented to our hospital with lethargy and right-sided weakness that started a week earlier. He also reported an episode of loss of consciousness lasting 5 minutes, which was not accompanied by jerking movements or gaze according to the witnesses, and the patient regained consciousness after 30 minutes. He did not report any headaches, dizziness, fever, or history of head trauma. He had a history of COVID-19, 3 months ago, which was complicated by pleural effusion and pneumothorax and led to ICU admission. He was discharged from the previous hospital 3 weeks prior to these presentations. Other than the mentioned conditions, his past medical history included ischemic heart disease for which he underwent coronary artery bypass grafting (CABG) 7 years ago. His current medications included aspirin and metoprolol.

Physical examination revealed an afebrile man who maintained eye contact but had limited verbal communication and was only able to obey two-step verbal commands following multiple repetitions. His motor strength was decreased in both right upper and lower limbs (4/5) and he had a right upward plantar reflex. He was unable to walk due to generalized weakness.

Initial laboratory results showed leukocytosis (17,600/µl, normal 4000–10000), anemia (11.4 g/dl, normal 14–18), thrombocytopenia (108,000/µl, normal 140,000–440,000), hyponatremia (125 mEq/l, normal 136–145), increased erythrocyte sedimentation rate (ESR 36 mm/hour, normal < 20) and normal C-reactive protein (CRP < 6 mg/l, normal < 6).

**Fig. 2:**
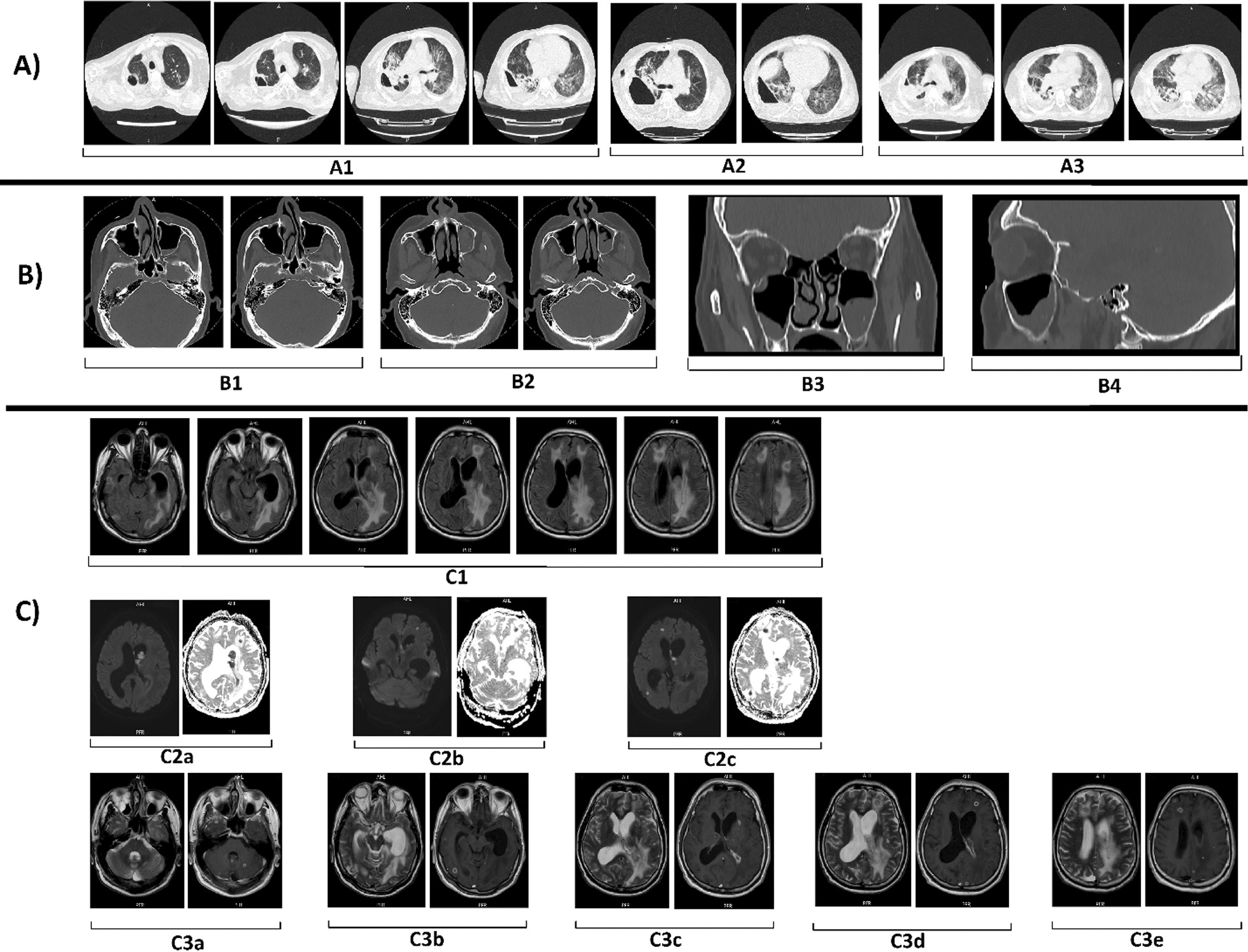
**A** Computerized tomography (CT) scan of the lungs at admission day (A1), 11 days (A2), and 26 days (A3) later. There is a right-sided loculated hydropneumothorax connected to a parenchymal abscess. Lung parenchyma shows widespread ground glass with some reticular opacities bilaterally. **B** Paranasal sinus CT scan on day 5 of admission shows mucosal thickening in maxillary sinuses, air bubbles, and air-fluid level in left maxillary sinuses in favor of acute sinusitis (B1). Punctuate, linear, and irregular calcifications in the axial (B2 left plane), coronal (B3), and sagittal (B4) views and a round nonfluid collection (B2 right plane) are visible in the left maxillary sinus, together suggesting an underlying fungus ball with superimposed acute sinusitis. **C** Brain MRI on day 2 of admission. In the upper row (C1) in the fluid-attenuated inversion recovery sequences of MRI (FLAIR) there are multiple high-signal round mass-like lesions throughout the brain parenchyma with cortico-subcortical junction distribution and with significant peripheral vasogenic edema. There is high signal material in the left lateral ventricle and asymmetrical dilation of the ventricles with intraventricular septal deviation to right (noncommunicating hydrocephalus). In the middle row, diffusion-weighted imaging (DWI) (C2a–c left planes) and apparent diffusion coefficient (ADC) (C2a–c right planes) sequences, almost all the mass-like lesions as well as the intraventricular material have shown diffusion restriction. In the bottom row, T2 (C3a–e left planes) and contrast-enhanced T1 MRI sequences (C3a–e right planes), the mass-like lesions have ring enhancement, and the intraventricular collection has shown thick enhancement in close relationship to the ependymal lining of the left lateral ventricle. All the above findings are in favor of multiple brain abscesses and ventriculitis with significant vasogenic brain edema and noncommunicating hydrocephalus

MRI of the brain revealed multiple high signal lesions with significant peripheral enhancement in the subcortical and deep white matter in both hemispheres suggestive of brain abscesses. Mucosal thickening of paranasal sinuses in favor of chronic sinusitis was also observed (Fig. 2C). CT scan of the paranasal sinuses showed mucosal thickening in both maxillary sinuses. It also revealed punctuate and irregular calcifications, fluid level, and air bubbling in the left maxillary sinus suggestive of acute sinusitis superimposed on a fungus ball (Fig. 2B). CT scan of the chest revealed loculated hydropneumothorax connected to a large parenchymal abscess in the right lower lobe (Fig. 2A). Echocardiography was performed to assess for possible endocarditis, which revealed no vegetations.

Empiric therapy was started with cotrimoxazole, vancomycin, cefepime, metronidazole, and anti-TB drugs. Amphotericin B was added to the drug regimen due to a suspected fungal infection. Blood cultures, viral hepatitis panel, and HIV antibody came back negative. IgG for *Toxoplasma gondii* was positive but IgM was negative. Sputum smear and culture andPCR of the pleural fluid came back negative for tuberculosis, and anti-TB drugs were discontinued. Smear and culture of the cerebrospinal fluid (CSF) did not show any organisms. The patient was scheduled for stereotactic biopsy and drainage of one of the brain abscesses. Drained fluid from the abscess was sent for microbiological evaluation.

The patient’s neurological symptoms did not improve, and his consciousness deteriorated. He was intubated and underwent mechanical ventilation. Repeat imaging of the brain showed multiple hypodense lesions with rim enhancement in frontal and occipital lobes with surrounding vasogenic edema suggestive of brain abscesses. One of the lesions had developed an intraventricular part, causing dilation of the left ventricular frontal and temporal horns and midline shift. Evidence of ventriculitis in the left ventricular occipital horn was also observed, and opacifications were seen in bilateral mastoid air cells. The patient was scheduled for a ventriculoperitoneal shunt placement to relieve the obstructive hydrocephalus caused by the intraventricular abscess. PCR of the pleural fluid revealed *Klebsiella pneumonia*, hence, the antibiotic regimen was changed to meropenem, colistin, and linezolid based on the resistance profile. Eventually, PCR of the brain abscess fluid revealed *Aspergillus fumigatus* as the culprit organism, and antifungal therapy was changed to voriconazole.

Despite appropriate medical therapy, the patient’s condition did not improve. Forty days after admission, he entered cardiac arrest and could not be resuscitated.

## Discussion

We report the first case of brain and intramedullary abscesses caused by *Pseudomonas aeruginosa* and a rare case of brain abscesses caused by *Aspergillus fumigatus* in two post-COVID-19 patients. A few studies have reported invasive cerebral infections with unusual organisms in patients with COVID-19 (Table 1). Two studies reported fungal brain abscesses associated with trichosporonosis and phaeohyphomycosis, both in patients with diabetes[22, 23]. This suggests that COVID-19 infection can predispose patients to unusual infections with unusual pathogens. There are multiple reports of invasive rhino–orbital–cerebral mucormycosis in patients with COVID-19, most of them being diabetic [24]. COVID-19 has been associated with the development and exacerbation of diabetes mellitus (DM) [25]. Higher blood sugar levels are closely associated with worse outcomes in patients with COVID-19, as patients with diabetes have impaired phagocytic activity, T cell function, neutrophil chemotaxis, and disrupted innate and adaptive immunity [26–28]. COVID-19 itself is also associated with immunosuppression and disrupts the activity of both innate and adaptive immune systems [29]. One study reported decreased number and impaired function of T lymphocytes and NK cells in hospitalized patients with COVID-19, which were more prominent in critically ill patients [30]. Our first patient did not have a history of DM but had elevated FBS and HbA1c levels suggesting undiagnosed DM. She also had high blood sugar levels throughout her admission, ranging from 176 to 435 mg/dl (normal 70–115). The relative immunocompromised state caused by her DM and COVID-19 may have predisposed her to the invasive *P. aeruginosa* infection with a poor prognosis. In addition, she received corticosteroid therapy for her COVID-19 infection, which may have contributed to her immunosuppression.

**Table 1:** Brain abscesses caused by unusual pathogens in patients with COVID-19

Authors	Year	Patient gender	Patient age	Comorbidities	Pathogen	Outcome
Laiq *et al.* [22]	2022	Female	73	Hypertension, DM	*Fonsecaea*	Death
Samaddar *et al.* [23]	2022	Male	55	Hypertension, DM	*Trichosporon dohaense*	Recovery
Gupta *et al.* [32]	2021	Male	62	DM	*Aspergillus fumigatus*	Recovery
De Villiers De La Noue *et al.* [31]	2021	Not mentioned	60	None	*Aspergillus fumigatus*	Recovery
Shahab *et al.* [42]	2021	Male	59	DM	Not detected (probably fungal)	Recovery

Our second patient had a recent history of severe COVID-19 leading to ICU admission. The severe COVID-19-induced immunosuppression might have made him susceptible to invasive aspergillosis. There have been two previous reports of *Aspergillus* brain abscesses in patients with COVID-19. Similar to our second patient, both of these patients had a history of recent ICU admission due to COVID-19 [31, 32]. Corticosteroid therapy, intubation and mechanical ventilation, and ongoing inflammation in ICU patients are risk factors that predispose them to invasive fungal infections [33].

The reported fungal brain abscesses following COVID-19 were mostly associated with contiguous spread from paranasal sinus involvement. In our second patient, imaging showed evidence of sinusitis, which suggests that the sinuses were the primary source of *Aspergillus* infection. However, a study reported a case of a brain abscess caused by *A. fumigatus* in a diabetic patient with COVID- 19 without sinonasal involvement, which had occurred through hematogenous spread from the lung. The authors suggested that a brain MRI should be obtained in patients with COVID-19 presenting with neurological symptoms either during their disease or after recovery to rule out brain abscesses, even without any evidence of rhino-orbital involvement [32]. In our first patient, a CT scan of the head showed no involvement of paranasal sinuses or orbits. In another study, *P. aeruginosa* caused malignant external otitis in a 65-year-old man with uncontrolled DM, which progressed to involve the temporal bone and skull base and caused multiple cranial nerve palsies. The patient’s clinical course was complicated by COVID-19 infection, and he passed away [34]. This points out that concomitant DM, COVID-19, and *P. aeruginosa*infection are associated with a poor prognosis. Our first patient had no otorrhea or otalgia and there was no evidence of ear infection in the physical examination or brain imaging. These findings suggest that *P. aeruginosa* has likely spread to the brain through a hematogenous route in our first patient, which justifies the coexistence of an intramedullary abscess.

There have been a few reports of *Pseudomonas *spp. causing complications in patients with COVID-19 (Table 2). *Pseudomonas aeruginosa* caused multiple skin abscesses on the forearm of an otherwise immunocompetent patient with COVID-19 [35]. In another study, *Pseudomonas putida*, an opportunistic bacteria causing infections in immunosuppressed patients, caused an exacerbation of bronchiectasis in a 70-year-old patient with COVID-19 who was otherwise immunocompetent. COVID-19 has been associated with worse outcomes in patients with bronchiectasis [36]. Our first patient was the first case of COVID-19 complicated by *P. aeruginosa* brain abscesses. Gregorova *et al.* reported a patient with COVID-19 that contracted recurring ventilator-associated pneumonias (VAP) with antibiotic-resistant *P. aeruginosa*, which led to a lengthy hospital stay in the intensive care unit. They speculated that COVID-19 infection resulted in a heightened immune system response that was further stimulated by the recurring *P. aeruginosa* infections. This led to bystander activation of T cells specific for antigens unrelated to either SARS-CoV2 or *P. aeruginosa*, which caused the more severe disease and complications experienced by this patient [37].

**Table 2:** Complications caused by *Pseudomonas *spp. in patients with COVID-19

Authors	Year	Patient gender	Patient age	Comorbidities	Disease	Outcome
Nelwan *et al.* [35]	2021	Female	53	None	Skin abscesses	Recovery
Silveira *et al.* [34]	2020	Male	65	Hypertension, DM	Malignant external otitis, cranial nerve palsies	Death
Gregorova *et al.* [37]	2020	Male	50	None	Recurring VAP	Recovery
Georgakopoulou *et al.* [36]	2021	Female	70	Hypertension, asthma, hypothyroidism, sleep apnea	Exacerbation of bronchiectasis	Recovery

Unfortunately, *P. aeruginosa* has been demonstrating increasing antibiotic resistance. Perez *et al.* recovered *P. aeruginosa* isolates from hospitalized patients during the COVID-19 pandemic and found a high resistance rate among them, probably caused by the production of New Delhi metallo-β-lactamases (NDMs) [38]. Liu *et al.* evaluated critically ill hospitalized patients with COVID-19 with bacterial infection and found that *P. aeruginosa* was the pneumonia organism that most commonly developed antimicrobial resistance, acquiring resistance to many broad-spectrum beta-lactam/beta-lactamase inhibitors, third-generation cephalosporins, and sometimes carbapenems [8]. However, another study found that *P. aeruginosa* isolates obtained from blood cultures and endotracheal tube aspirate cultures of patients with COVID-19 were 90% susceptible to imipenem [9]. PCR analysis of the *P. aeruginosa* strain in our first patient did not reveal the existence of any antibiotic resistance genes. However, she did not respond to extensive antibiotic therapy.

The most recent Infectious Diseases Society of America (IDSA) guidelines recommend voriconazole as the choice treatment for central nervous system (CNS) aspergillosis, and liposomal amphotericin B should be given only if the patient does not respond to treatment [39]. Both of the previously reported cases of *Aspergillus* brain abscesses in patients with COVID-19 responded to voriconazole [31, 32]. However, our second patient did not respond to either medication. This could be a result of his weakened immune system or the development of antifungal resistance in the pathogen. Antifungal resistance has been reported in previous cases of aspergillosis in patients with COVID-19 [40, 41].

## Conclusion

The immune dysregulation induced by COVID-19 and its treatments can predispose patients, especially immunosuppressed ones, to bacterial and fungal infections with unusual and opportunistic pathogens in unusual sites, including the central nervous system. *Pseudomonas aeruginosa* and *Aspergillus fumigatus* should be considered as crucial causes of central nervous system infection in any patient with COVID-19 presenting with neurological symptoms and evidence of brain abscess in imaging, regardless of sinonasal involvement. These patients should get started on appropriate antimicrobial therapy immediately, as any delay in diagnosis or treatment will be associated with adverse outcomes. Because of the increasing rate of antimicrobial resistance, antimicrobial therapy should be tailored according to the pathogen’s antibiotic resistance profile.

## Data Availability

Data resulted from this study are available from the corresponding author on reasonable request.

## Abbreviations

COVID-19: Coronavirus disease 2019
SARS-CoV-2: Severe acute respiratory syndrome coronavirus 2
ICU: Intensive care unit
PCR: Polymerase chain reaction
IDSA: Infectious diseases society of America
DM: Diabetes mellitus
MRSA: Methicillin-resistant *Staphylococcus aureus*
CAPA: COVID-19-associated pulmonary aspergillosis
CT: Computerized tomography
MRI: Magnetic resonance imaging
PMN: Polymorphonuclear
CABG: Coronary artery bypass grafting
CSF: Cerebrospinal fluid
VAP: Ventilator-associated pneumonia
NDMs: New Delhi metallo-β-lactamases
LDH: Lactate dehydrogenase
FBS: Fasting blood sugar
ESR: Erythrocyte sedimentation rate
CRP: C-reactive protein
AST: Aspartate aminotransferase
ALT: Alanine aminotransferase
CNS: Central nervous system
